# Dual improvement of cognitive function and auditory ability in elderly patients with hearing impairment by transcranial direct current stimulation-assisted auditory rehabilitation training

**DOI:** 10.3389/fnagi.2025.1591496

**Published:** 2025-09-01

**Authors:** Ying Zhou, Haolun Han, Xiaoli Zhang, Yiyan Zhang, Wenbo Duan, Liyun Su, Baowei Li, Zhezhe Sun, Lei Wang, Gang Wang

**Affiliations:** Department of Otolaryngology, The Ninth Medical Center of Chinese PLA General Hospital, Beijing, China

**Keywords:** transcranial direct current stimulation, auditory rehabilitation training, elderly hearing loss, hearing impairment, cognitive function, auditory ability

## Abstract

**Objective:**

To analyze the dual improvement effects of transcranial direct current stimulation (tDCS)-assisted auditory rehabilitation training on cognitive function and auditory ability of elderly patients with hearing impairment.

**Methods:**

100 cases of elderly patients with hearing impairment admitted to our hospital between January 2020 and January 2025 were prospectively selected as study subjects. The patients were divided into sham tDCS group (*N* = 50) and tDCS group (*N* = 50) according to the randomized numeric table method. All patients received conventional auditory rehabilitation training, and were intervened for 1 month, 3 times/week, 1 h each time. tDCS was given to patients in both groups before conventional auditory rehabilitation training, patients in the tDCS group underwent dual-site sequential high-definition tDCS stimulation, and patients in the sham tDCS group used sham dual-site sequential high definition tDCS stimulation. The main clinical assessments included hearing thresholds, Hearing Handicap Inventory for the Elderly-Screening (HHIE-S), Montreal Cognitive Assessment Scale (MoCA) and Mini-Mental State Examination (MMSE), Communication Performance Assessment (CPA), Personal Report of Communication Apprehension (PRCA-24), and 36-item Short-Form Health Survey (SF-36) scores of the patients in the two groups before and after the treatment. The correlation between hearing threshold, HHIE-S and MoCA and MMSE scores were analyzed by Pearson correlation coefficient.

**Results:**

There were no significant differences between the two groups in terms of age, gender, BMI, degree of hearing loss, education level, smoking and drinking habits, laboratory indicators [FBG, ALP, ALT, AST, TC, TG, HDL-C, LDL-C], comorbidities, and family history of hearing loss (all *p* > 0.05). The hearing thresholds and HHIE-S scores of patients in both groups after treatment were significantly lower than those before treatment (both *p* = 0.001), and the hearing thresholds and HHIE-S scores of patients in the tDCS group after treatment were significantly lower than those in the sham tDCS group (*p* < 0.001 and *p* = 0.002, respectively). The MoCA and MMSE scores of patients in both groups were significantly higher than those before treatment (both *p* < 0.001), and the MoCA and MMSE scores of patients in the tDCS group were significantly higher than those in the sham tDCS group after treatment (*p* = 0.048 and *p* = 0.038, respectively). Hearing thresholds and HHIE-S were negatively correlated with MoCA and MMSE scores in elderly patients with hearing impairment (all *p* < 0.05). Bootstrap mediation analysis suggests that changes in hearing impairment may partially mediate improvements in cognitive function. After treatment, the total CPA and SF-36 scores of all patients were higher than before treatment, and the total PRCA-24 score was lower than before treatment (*p* < 0.05). The CPA and SF-36 total scores of the patients in the tDCS group were higher than those in the sham tDCS group after treatment (*p* = 0.012 and *p* = 0.007, respectively), and the differences in the PRCA-24 total scores of the two groups were not statistically significant when compared with each other after treatment (*p* = 0.248).

**Conclusion:**

Transcranial direct current stimulation-assisted auditory rehabilitation training may improve the cognitive and auditory functions of elderly patients with hearing impairment and enhance the quality of life of patients.

## 1 Introduction

Geriatric hearing loss is defined as a decline in auditory function with age, caused by aging and degenerative lesions of the auditory organs (Slade et al., 2020; Lazzarotto et al., 2018). According to statistics, hearing loss has become the third most important cause of disability disorders in the elderly worldwide (Yang and Cosetti, 2016), and geriatric hearing impairment affects about one-third or so of people over 60 years of age globally, and will reach 900 million people with geriatric hearing impairment by 2050 (Sprinzl and Riechelmann, 2010). A growing body of research confirms the strong link between hearing and cognitive function (Johnson et al., 2021). Hearing loss affects older adults not only in speech perception and speech recognition, but also negatively affects cognitive function (Samelli et al., 2022).

Hearing impairment has been shown to be an independent risk factor for cognitive dysfunction. The risk of dementia in people with mild, moderate, and severe hearing impairment is 2, 3, and 5 times higher than that of people with normal hearing, respectively. This risk is also positively correlated with the duration of the disease. The longer the duration of the disease, the more severe the hearing impairment. Consequently, the chances of cognitive impairment increase and the degree of cognitive impairment becomes more severe (Lin and Ferrucci, 2012; Yuan et al., 2018; Loughrey et al., 2018).

At present, there is no definite treatment plan for geriatric hearing impairment, and hearing rehabilitation means such as hearing aids and cochlear implants are generally used, which are affected by many factors such as patients’ age, level of residual hearing, type of hearing loss, cognitive level, etc., and there is a significant difference in the intervention effect of different means of hearing rehabilitation (Tsai Do et al., 2024). Auditory rehabilitation training is a product of the integrated medical-educational-social model, and its proper use can maximize the improvement of patients’ ability to communicate and express themselves and their ability to hear, and alleviate the adverse effects of hearing abnormalities (Ubrig et al., 2019; Stropahl et al., 2020). Previous studies have shown that the effect of auditory training on acceptable noise levels (ANL) in older adults with hearing loss is similar to that of speech noise scores, providing hope for those for whom hearing aids are not appropriate; noisy speech training at good and poor signal-to-noise ratios has a positive effect on ANL, noisy speech (Kannada), and noisy conversation (COSI) (Nakshathri and Mohan, 2022). Auditory training for adults with high-frequency hearing loss improves figure-ground hearing skills in speech, temporal sequencing and discrimination, and communication in noisy environments (Santos et al., 2014).

Transcranial direct current stimulation (tDCS) is a non-invasive neuromodulation technique applied to the scalp that alters cortical neural activity by applying low-intensity direct current generated by electrode pads of different polarities (Nitsche and Paulus, 2000). tDCS induces prolonged changes in cerebral excitability and promotes cerebral plasticity, and it is an important direction of research in the field of neurorehabilitation (Fregni et al., 2021). In recent years, tDCS has been used in a number of fields such as post-stroke aphasia, Parkinson’s, depression, schizophrenia, postoperative pain, etc. (Cai et al., 2019; Burton et al., 2023). A study by Mori et al. (2016) scholars reported that a 13-year-old female patient who presented with bilateral hearing deficits due to brainstem encephalitis at the age of 6 years had a sustained and significant improvement in maximal speech recognition after tDCS treatment. Most previous studies have confirmed that repetitive tDCS of the auditory cortex does not appear to adversely affect hearing or cognition and may modestly improve hearing in noise (Leaver, 2025) and that stimulation of the right dorsolateral prefrontal cortex significantly improves tinnitus perception, with greater improvement in female tinnitus patients (Jacquemin et al., 2021).

Although large-scale evidence-based results are currently lacking, a few pilot trials have suggested the synergistic potential of tDCS with auditory-related training. For example, Chow et al. (2021) used left DLPFC anodal tDCS combined with personalized music listening in 14 healthy elderly subjects and observed short-term improvements in pure tone thresholds, speech recognition in noise, and working memory. Another randomized double-blind crossover study (Ladeira et al., 2011) demonstrated in 20 subjects that 4 × 1 HD-tDCS applied to the auditory cortex could improve central auditory temporal processing in a polarity-dependent manner. These pilot results support the exploration of “bidirectional auditory-cognitive benefit” mechanisms in this study and provide reference for parameter selection.

Currently, there are few studies on tDCS-assisted auditory rehabilitation training on cognitive function and auditory ability in patients with senile hearing impairment. The aim of this study is to analyze the dual improvement effects of tDCS-assisted auditory rehabilitation training on cognitive function and auditory ability of elderly patients with hearing impairment, with the aim of providing new references for the clinical treatment of elderly patients with hearing impairment.

## 2 Materials and methods

### 2.1 Study population

Prospectively selected 136 elderly patients with hearing impairment admitted to our hospital between January 2020 and January 2025, 115 patients were screened according to the inclusion and exclusion criteria, of which 15 refused to participate and were excluded, and 100 patients were finally included as study subjects. The patients were divided into sham tDCS group (*N* = 50) and tDCS group (*N* = 50) according to the random number table method. All patients received conventional auditory rehabilitation training, all intervened for 1 month, 3 times/week, 1 h each time. tDCS was given to patients in both groups before conventional auditory rehabilitation training, patients in the tDCS group underwent dual-site sequential High-Definition tDCS stimulation, and patients in the sham tDCS group were given sham dual-site sequential High-Definition tDCS stimulation, and no one withdrew in the middle of the study. The study was reviewed and approved by the Ethics Committee of our hospital and complied with the Declaration of Helsinki.

### 2.2 Inclusion and exclusion criteria

#### 2.2.1 Inclusion criteria

(1) Progressive sensorineural deafness in both ears without obvious triggers, all wearing hearing aids for >6 months, all hearing aids were in-the-ear and binaural; (2) age 60 years and above; (3) no intellectual disability, no history of psychiatric disorders, and capable of normal verbal or written communication (Lin, 2024).

#### 2.2.2 Exclusion criteria

(1) Hearing loss caused by other factors such as trauma, drug toxic hearing loss, hearing loss caused by otitis media, congenital malformations, Meniere’s disease, acoustic neuroma, and autoimmune hearing loss; (2) severe systemic diseases, history of psychiatric disorders, and inability to comprehend the content of the questionnaire; (3) combination of cardiac, hepatic, renal, and other important organ dysfunctions (well-controlled hypertension/diabetes permitted).

In accordance with the principle of randomization, 100 patients were randomly divided into the control group and the study group with the ratio of 1:1 using the random number table method after the patients signed the informed consent form. The specific methods were as follows: 100 Elderly patients with hearing impairment (numbers 1–100) were divided into 2 groups by the principal investigator (PI). The subjects corresponding to these 50 numbers were assigned to 1 group, and the remaining subjects were assigned to another group. The randomization schedule was concealed in a locked cabinet accessed only by the PI. Hence, patients were blind regarding the type of transcranial direct current stimulation they received (real or sham). Neither the participants nor the researchers assessing outcomes were aware of the interventions administered. Subjects were blinded to the experimental assumptions and were not allowed to discuss their experience during the intervention with the researchers or other subjects involved.

### 2.3 Routine auditory rehabilitation training

All patients received auditory rehabilitation training (Barcroft et al., 2016), the training room was a soundproof room, the patients were divided into 5 groups, 10 people in each group, and each group was guided by 2 interventionists for training. Auditory training included: ① Finding the sound source: patients closed their eyes while sounds were given from different directions (clapping, whistling, playing music), and patients judged the direction; ② Listening to rhythm training: interventionists rhythmically knocked on the table, and patients repeated the rhythm; ③ Repeating sentences: playing a sentence for patients to repeat; ④ Finding differences: playing two similar sentences for patients to identify differences; ⑤ Word recognition training: playing different categories of words (animals, food, plants) for categorization; ⑥ Chinese character repetition order: listening to a set of words with corresponding numbers and repeating in numerical order; ⑦ Identifying sounds: patients closed eyes while different people made sounds for identification; ⑧ Points to note: reducing external cues during training, choosing familiar everyday sounds, focusing on communication and motivation, setting appropriate achievable milestones. All patients were intervened for 1 month, 3 times/week for 1 h. This programme was administered by 2 specifically trained physical therapists, both of whom were blinded.

### 2.4 Transcranial direct current stimulation

As previously described (Cardon et al., 2022), all patients were treated with a High-Definition tDCS instrument (Soterix Medical), with 15 min of stimulation of two regions at a time for a total stimulation time of 30 min. Three times/week, each time at least 1 day apart, for a total of 1 month of treatment. Electrode positioning was performed according to the 10/20 international system for electroencephalogram (EEG) electrode placement, with electrodes placed at the right dorsolateral prefrontal cortex (rDLPFC) and left temporal area (LTA). The 4 × 1 HD-tDCS montage consisted of central anodes placed at F4 (rDLPFC) and CP5 (LTA), with surrounding cathodes at F2, F6, FC4 and AF4 (rDLPFC) and C5, TP7, CP3 and P5 (LTA). A constant current of 2 mA was applied at each site for 15 min, with a fade-in and fade-out time of 20s. The order of stimulation (rDLPFC-first vs. LTA-first) was randomized for each participant. Second site started 5 min after first; electrodes repositioned during interval. For the sham stimulation, constant current was applied for the first 20s followed by 0 mA for the remainder of the session. Direct current was applied via sintered Ag/AgCl ring electrodes with an inner radius of 6 mm and outer radius of 12 mm and delivered via a battery-driven 1 × 1 tDCS low-intensity stimulator and 4 × 1 multichannel stimulation adaptor (Soterix Medical Inc.). Ring electrodes were stabilized using HD-electrode holders anchored in a Soterix Medical HD-cap and filled with EEG electrode gel (Neurax) following the guidelines for 4 × 1 HD-tDCS stimulation.

### 2.5 Clinical assessment

Auditory function, cognitive function, communication ability and fear of communication status, and quality of life were assessed in the two groups before and 1 month after the intervention, respectively.

#### 2.5.1 Auditory ability

① Hearing threshold: Apply pure tone audiometer LS0402A to conduct pure tone hearing threshold test at 4 frequencies of 500, 1,000, 2000 and 4,000 Hz, with background noise <30 dB, and calculate the average value (4FA). ② Degree of hearing impairment: The Hearing Handicap Inventory for the Elderly-Screening (HHIE-S) contains emotional and social scenarios, with 10 questions, scored as 0, 2, or 4, and a total score of 40 points. A score of ≤ 8 is considered no hearing impairment and vice versa, 10–24 is considered mild to moderate, and 26–40 is considered severe.

#### 2.5.2 Cognitive function

① Montreal Cognitive Assessment Scale (MoCA) contains 7 aspects of language, naming, attention, memory and delayed memory, visuospatial and executive function, abstraction, orientation, 1 point for full compliance, 0 points for the opposite, the whole test is controlled within 10 min, a full score of 30 points, and if the patient’s literacy level is ≤ 12 years, an additional 1 point. ≥26 points for normal cognitive function, and vice versa for cognitive dysfunction, 19 ~ 25 points for mild, 10 ~ 18 points for moderate, <10 points for severe. ② Mini-mental state examination (MMSE) includes 5 aspects, including language (9 points), orientation (10 points), memory (3 points), recall (3 points), attention and calculation (5 points), with a score of 30 points, 27–30 points for normal cognitive function, <27 points for cognitive dysfunction, 21 ~ 26 points for mild, 10 ~ 20 points for moderate, and 0 ~ 9 points for severe.

#### 2.5.3 Communication performance

The communication performance assessment (CPA) was used to assess communication performance, with a total of 30 items, including 8 items of self-assessment, 4 items of interpersonal communication assessment, 13 items of the impact of hearing abnormality on social interaction, and 5 items of the impact of impaired hearing on occupations, and a question-and-answer format was used, with each item divided into 5 grades, and a score ranging from 30 to 150 points, with a high score representing a high level of communication ability.

#### 2.5.4 Fear of communication

The personal report of communication apprehension (PRCA-24) was used to assess the patient’s discomfort during communication. The PRCA-24 contains four scales: “two-person, group, meeting, and public,” which scores the degree of fear in two-person communication, multi-person communication in a group, attending a meeting, and public speaking, etc. Each subscale contained 6 entries and was scored on a 5-point scale, with a value of 6–30 points from very satisfied to very dissatisfied, and a total score of 24–120 points. A high score represents a high degree of fear, and a total score of >79 points indicates a high degree of fear, while a total score of 55–79 points indicates a moderate fear, and a total score of <55 points indicates a low degree of fear. The mean score of the scale was taken as the result.

#### 2.5.5 Quality of life

The Brief Healthy Living Conditions Scale (36-item short-form, SF-36) was used to contain 8 dimensions and 36 entries, which were physical functioning (10 items), somatic pain (2 items), physical functioning (4 items), affective functioning (3 items), social functioning (2 items), mental health (5 items), energy (4 items), general health status (5 items) and overall health (1 item). Each item is scored on a scale of 0 to 100, with higher scores indicating a higher quality of life.

### 2.6 Clinical data collection

Information on patient’s age, gender, BMI, degree of hearing loss, education level, smoking and drinking habits, comorbidities (hypertension, hyperlipidemia, diabetes mellitus), and family history of hearing loss were collected.

### 2.7 Blood biochemistry

Morning fasting venous blood and morning urine were collected from all study subjects by trained nurses according to uniform requirements and promptly sent to the laboratory department of the hospital where the study subjects had their physical examinations, and were analyzed using an ARCHITECT Ci8200 fully automated biochemistry-immunoanalyzer (ABBOTT Laboratories, AbbottPark, Illinois, USA) and the company’s standardized reagents. Company’s standardized reagents for testing. The main test items included: blood routine, urine routine, fasting blood glucose (FBG). Liver enzymes: alanine aminotransferase (ALT), aspartate aminotransferase (AST), alkaline phosphatase (ALP). Lipids: Total cholesterol (TC), Triglyceride (TG), Low density lipoprotein cholesterol (LDL-C) and High density lipoprotein cholesterol (HDL-C). Among them, serum bilirubin: serum total bilirubin (TBIL), direct bilirubin (DBIL), and indirect bilirubin (IBIL), are included in the items of blood biochemical tests.

### 2.8 Statistical analysis

Data were statistically analyzed and graphed using SPSS 27.0 statistical software (SPSS, Inc., Chicago, IL, USA) and GraphPad Prism 9.5.0 software (GraphPad Software Inc., San Diego, CA, USA). The Shapiro–Wilk test was used to test for normal distribution, and measurements that conformed to normal distribution were expressed as mean ± standard deviation. The primary analysis for all continuous outcomes was a two-way repeated-measures ANOVA with factors group (tDCS vs. sham) and time (pre vs. post). When the interaction was significant, Bonferroni-corrected paired- and independent-sample *t*-tests were used as planned post-hoc comparisons. Independent samples *t*-tests were used for baseline comparisons between groups, and paired samples *t*-tests for within-group pre-post comparisons. Measurement information that was not normally distributed was expressed as the median (interquartile spacing), and the Mann–Whitney U test was used for intergroup comparisons, and the Wilcoxon signed-rank test was used for intragroup pre- and post-intervention comparisons; counting information was expressed as the number of cases and percentages, and the chi-square test was used for intergroup comparisons. *p* was a two-sided test, and the difference was considered to be statistically significant at *p* < 0.05.

## 3 Results

### 3.1 Comparison of baseline data of subjects in the two groups

There was no significant difference between the two groups in terms of age, gender, BMI, degree of hearing loss, education level, smoking and drinking habits, laboratory parameters [FBG, ALP, ALT, AST, TC, TG, HDL-C, LDL-C], comorbidities (hypertension, hyperlipidemia, and diabetes mellitus), and family history of hearing loss (all *p* > 0.05), as shown in Table 1.

**Table 1:** Comparison of baseline characteristics.

Characteristics	Sham tDCS group (*N* = 50)	tDCS group (*N* = 50)	*p*-value
Sex (m/f)	29/21	27/23	0.687
Age (years)	67.08 ± 3.98	66.76 ± 4.15	0.695
BMI (kg/m^2^)	24.25 ± 2.06	24.56 ± 2.44	0.494
Degree of hearing loss (*N*, %)			
Mild (25 ~ 40 dB)	13 (26.00%)	11 (22.00%)	0.558
Medium (41 ~ 60 dB)	36 (72.00%)	36 (72.00%)	
Heavy (61 ~ 80 dB)	1 (2.00%)	3 (6.00%)	
Upper secondary/secondary education and above (*N*, %)	20 (40.00%)	18 (36.00%)	0.680
Smoking history (*N*, %)	23 (46.00%)	26 (52.00%)	0.548
Drinking history (*N*, %)	18 (36.00%)	20 (40.00%)	0.680
Comorbidity (*N*, %)			
High blood pressure	29 (58.00%)	27 (54.00%)	0.687
Hyperlipidemia	18 (36.00%)	21 (42.00%)	0.539
Diabetes	9 (18.00%)	12 (24.00%)	0.461
Biochemical index			
FBG (mmol/L)	5.86 ± 1.26	5.77 ± 1.43	0.733
ALP (U/L)	82.95 ± 5.36	83.25 ± 4.95	0.772
ALT (U/L)	18.61 ± 2.32	19.12 ± 2.84	0.325
AST (U/L)	22.58 ± 3.00	23.17 ± 3.15	0.340
TC (mmol/L)	4.77 ± 0.81	4.71 ± 0.85	0.737
TG (mmol/L)	1.26 ± 0.26	1.28 ± 0.32	0.745
HDL-C (mmol/L)	1.39 ± 0.29	1.37 ± 0.33	0.723
LDL-C (mmol/L)	2.67 ± 0.49	2.72 ± 0.45	0.533
Family history of hearing loss (*N*, %)	3 (6.00%)	5 (10.00%)	0.461

BMI, Body Mass Index; FBG, Fasting blood glucose; ALP, alkaline phosphatase; ALT, alanine aminotransferase; AST, aspartate aminotransferase; TC, Total cholesterol; TG, Triglyceride; HDL-C, High density lipoprotein cholesterol; LDL-C, Low density lipoprotein cholesterol.

### 3.2 Comparison of auditory function between the two groups of patients

We compared the auditory functions of the two groups of patients, including hearing thresholds and HHIE-S scores, and the results showed that the differences between the hearing thresholds and HHIE-S scores of the two groups before treatment were not statistically significant (both *p* > 0.05). After treatment, the hearing thresholds and HHIE-S scores of patients in both groups were significantly lower than those before treatment (both *p* < 0.001). The hearing thresholds and HHIE-S scores of the patients in the tDCS group were significantly lower than those in the sham tDCS group after treatment (*p* < 0.001 and *p* < 0.001, respectively), Two-way ANOVA revealed a significant group × time interaction for hearing threshold (F_1,98_ = 5.4, *p* = 0.022) and HHIE-S (F_1,98_ = 7.8, *p* = 0.006), indicating greater improvement in the tDCS group (see Table 2) (Supplementary Table S1). Supplementary Figure S1 illustrates the individual trajectories of change in both groups.

**Table 2:** Comparison of auditory function between the two groups.

Outcome measures	Timing	Sham tDCS group (*N* = 50)	tDCS group (*N* = 50)	*p*-value
Hearing threshold (dB)^a^	Pre-treatment	45.10 ± 5.93	44.60 ± 6.88	0.300^c^
	Post-treatment	38.84 ± 4.82	35.08 ± 4.13	< 0.001^c^
	*p*-value	< 0.001^b^	< 0.001^b^	
	Δ (Post − Pre)	−6.26 ± 3.21	−9.52 ± 3.58	< 0.001^c^
HHIE-S (points)^a^	Pre-treatment	23.48 ± 2.63	23.02 ± 3.01	0.417^c^
	Post-treatment	15.24 ± 2.65	13.00 ± 1.94	< 0.001^c^
	*p*-value	< 0.001^b^	< 0.001^b^	
	Δ (Post − Pre)	−8.24 ± 2.95	−10.02 ± 2.91	0.003^c^

HHIE-S, hearing handicap inventory for the elderly-screening. Values are mean ± SD. Δ = change score (post-treatment minus pre-treatment).

a Group × Time ANOVA interaction (F₁,₉₈ = 5.4, *p* = 0.022 for hearing threshold; F₁,₉₈ = 7.8, *p* = 0.006 for HHIE-S).

b Within-group paired *t*-test.

c Between-group independent *t*-test.

### 3.3 Comparison of cognitive function between two groups of patients

We compared the cognitive functions of the two groups of patients, including MoCA and MMSE scores, and the results showed that there was no statistically significant difference between the MoCA and MMSE scores of the two groups of patients before treatment (both *p* > 0.05). After treatment, the MoCA and MMSE scores of patients in both groups were significantly higher than those before treatment (both *p* < 0.001). The MoCA and MMSE scores of the patients in the tDCS group were significantly higher than those in the sham tDCS group after treatment (*p* = 0.048 and *p* = 0.038, respectively), Two-way ANOVA revealed a significant group × time interaction for MoCA (F_1,98_ = 4.8, *p* = 0.031) and MMSE (F_1,98_ = 6.5, *p* = 0.012), indicating greater improvement in the tDCS group (see Table 3).

**Table 3:** Comparison of cognitive function between the two groups.

Outcome measures	Timing	Sham tDCS group (*N* = 50)	tDCS group (*N* = 50)	*p*-value
MoCA (points)^a^	Pre-treatment	19.98 ± 2.65	20.04 ± 3.03	0.916^c^
	Post-treatment	21.84 ± 2.45	22.86 ± 2.63	0.048^c^
	*p*-value	< 0.001^b^	< 0.001^b^	
	Δ (Post − Pre)	1.86 ± 1.42	2.82 ± 1.30	< 0.001^c^
MMSE (points)^a^	Pre-treatment	21.26 ± 2.47	21.54 ± 2.08	0.513^c^
	Post-treatment	23.82 ± 2.72	24.98 ± 2.80	0.038^c^
	*p*-value	< 0.001^b^	< 0.001^b^	
	Δ (Post − Pre)	2.56 ± 1.58	3.44 ± 1.49	0.005^c^

MoCA, Montreal cognitive assessment; MMSE, mini-mental state examination. Values are mean ± SD. Δ = change score (post-treatment minus pre-treatment).

a Group × Time ANOVA interaction (F₁,₉₈ = 4.8, *p* = 0.031 for MoCA; F₁,₉₈ = 6.5, *p* = 0.012 for MMSE).

b Within-group paired *t*-test.

c Between-group independent *t*-test.

### 3.4 Correlation analysis of auditory function and cognitive function in elderly patients with hearing impairment

**Figure 1:**
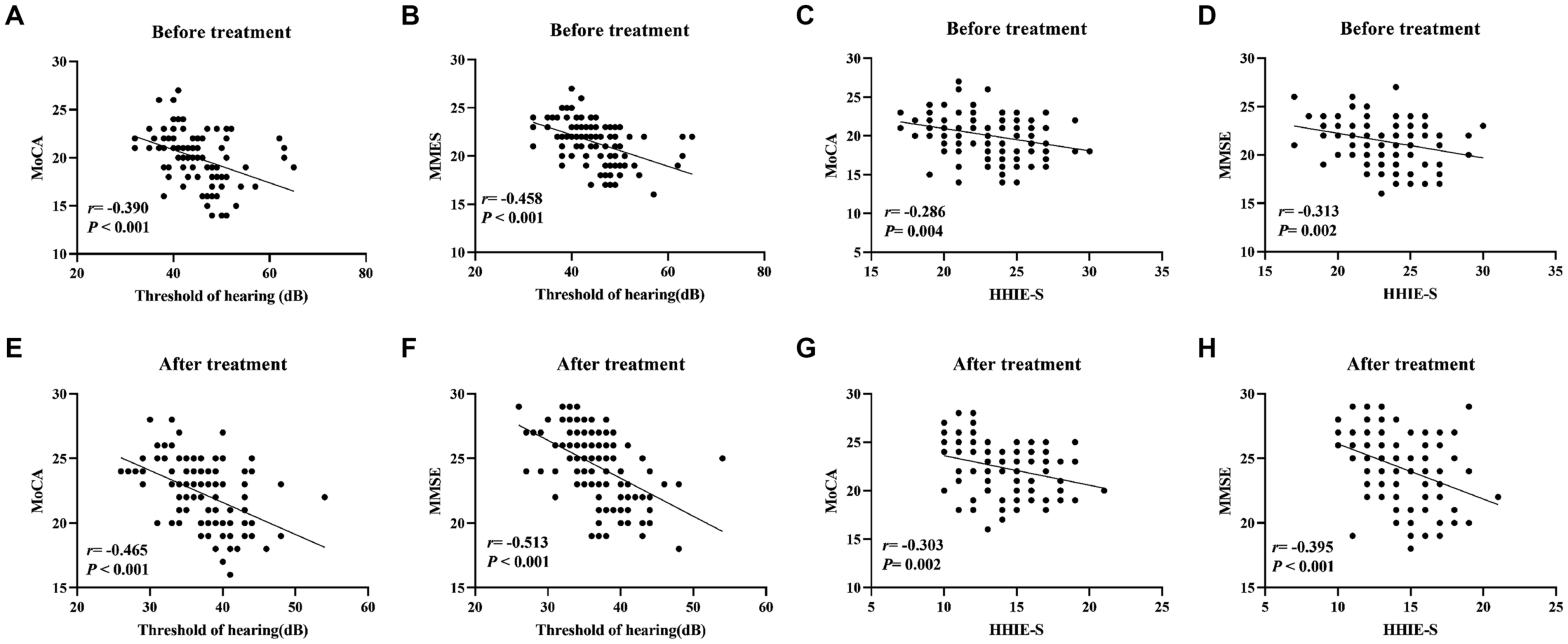
Correlation analysis of hearing thresholds, HHIE-S with MoCA and MMSE scores in elderly patients with hearing impairment. **(A–D)** Pearson was used to analyze the correlation between pre-treatment hearing thresholds, HHIE-S and MoCA and MMSE scores; **(E–H)** Pearson was used to analyze the correlation between post-treatment hearing thresholds, HHIE-S and MoCA and MMSE scores. r is the correlation coefficient. Differences were considered statistically significant at *p* < 0.05.

In order to investigate the relationship between auditory function and cognitive function in elderly patients with hearing impairment, we analyzed the correlation between hearing thresholds, HHIE-S and MoCA and MMSE scores by using Pearson correlation coefficient. The results of Pearson’s analysis showed that hearing thresholds, HHIE-S were negatively correlated with MoCA and MMSE scores of the elderly patients with hearing impairment (all *p* < 0.05, Figure 1).

### 3.5 Comparison of communication ability and fear of communication status and quality of life between the two groups of patients

The results showed that there was no statistically significant difference between the CPA, PRCA-24 and SF-36 scores of the two groups of patients before treatment (all *p* > 0.05). After treatment, the total scores of CPA and SF-36 of all patients were higher than before treatment, and the total score of PRCA-24 was lower than before treatment (*p* < 0.05). The CPA and SF-36 total scores of the patients in the tDCS group were higher than those in the sham tDCS group after treatment (*p* = 0.012 and *p* = 0.007, respectively), and the difference in the PRCA-24 total scores between the two groups after treatment was not statistically significant (*p* = 0.248), two-way ANOVA revealed a significant group × time interaction for CPA (F_1,98_ = 8.2, *p* = 0.005) and SF-36 (F_1,98_ = 7.1, *p* = 0.009), but not for PRCA-24 (F_1,98_ = 1.3, *p* = 0.257), as shown in Table 4. Sequence-order subgroup analysis (Supplementary Table S2) revealed no significant differences between rDLPFC-first and LTA-first stimulation orders (*p* > 0.05).

**Table 4:** Comparison of communication ability and communication fear status and quality of life between the two groups.

Outcome measures	Timing	Sham tDCS group (*N* = 50)	tDCS group (*N* = 50)	*p*-value
CPA (points)^a^	Pre-treatment	70.12 ± 5.67	71.58 ± 6.36	0.241^c^
	Post-treatment	80.50 ± 6.93	84.10 ± 7.05	0.012^c^
	*p*-value	< 0.001^b^	< 0.001^b^	
	Δ (Post − Pre)	10.38 ± 4.15	12.52 ± 4.38	0.013^c^
PRCA-24 (points)	Pre-treatment	70.44 ± 7.27	69.50 ± 8.93	0.565^c^
	Post-treatment	60.20 ± 7.51	58.52 ± 6.95	0.248ᶜ
	P-value	< 0.001^b^	< 0.001^b^	
	Δ (Post − Pre)	−10.24 ± 4.82	−10.98 ± 5.12	0.458^c^
SF-36 (points)^a^	Pre-treatment	75.86 ± 6.97	76.16 ± 7.03	0.831^c^
	Post-treatment	82.18 ± 4.92	85.10 ± 5.70	0.007^c^
	*p*-value	< 0.001^b^	< 0.001^b^	
	Δ (Post – Pre)	6.32 ± 3.85	8.94 ± 4.23	0.002^c^

CPA, communication performance assessment; PRCA-24, personal report of communication apprehension; SF-36, 36-item short-form. Values are mean ± SD. Δ = change score (post-treatment minus pre-treatment).

a Group × Time ANOVA interaction (F₁,₉₈ = 8.2, *p* = 0.005 for CPA; F₁,₉₈ = 1.3, *p* = 0.257 for PRCA-24; F₁,₉₈ = 7.1, *p* = 0.009 for SF-36).

b Within-group paired *t*-test.

c Between-group independent *t*-test.

### 3.6 Safety evaluation

We counted the adverse reactions in the treatment process of the two groups of patients and found that all patients did not have any obvious adverse reactions during the treatment process. As shown in Supplementary Table S3, tingling sensation occurred in 6/50 patients in the HD-tDCS group and 4/50 in the sham group; mild headache in 3/50 in the HD-tDCS group and 2/50 in the sham group; none withdrew due to adverse events.

### 3.7 Order effect analysis

Order-effect analysis (Supplementary Table S2) showed no significant rDLPFC-first versus LTA-first differences for any primary or secondary outcome (all *p* > 0.05), suggesting that the stimulation sequence did not influence treatment effects. Full ANOVA outputs (*F*, p, partial *η*^2^) for every endpoint are provided in Supplementary Table S4.

## 4 Discussion

The main causes of auditory decline in patients with age-related hearing loss are related to the aging of the peripheral auditory system and the aging of the central auditory system, and hearing loss may lead to a reduction in cognitive resources used for complex cognitive processes, such as executive ability, memory capacity, and information processing speed (Sharma et al., 2021). Aging-related hearing loss and vestibular decline can affect cognitive function, and hearing loss is a risk factor for mild cognitive impairment and dementia (Ghisletta et al., 2023). It can be hypothesized that age-related deafness is associated with cognitive function. The aim of this study was to analyze the role of tDCS-assisted auditory rehabilitation training on cognitive function and auditory ability of elderly patients with hearing impairment, with a view to providing a new reference for the clinical treatment of elderly patients with hearing impairment.

The results of this study showed that the hearing thresholds and HHIE-S scores of patients in both groups were significantly lower after treatment than before treatment, and the hearing thresholds and HHIE-S scores of patients in the tDCS group were significantly lower than those in the sham tDCS group after treatment. It is suggested that tDCS-assisted auditory rehabilitation training may have a positive effect on the auditory function of patients with senile hearing impairment. The reason for this analysis is that patients with senile hearing impairment have delayed recovery of neural excitability, impaired neural synchronization, and the auditory system is unable to accurately encode the temporal characteristics of speech, so that the target speech is susceptible to masking by noise (Jafari et al., 2019). Auditory training stimulates the auditory pathway through searching for sound sources, listening to tapping, repeating sentences, identifying sounds and other training to strengthen the processing capacity of the auditory center, correct the auditory system’s dysregulation of sound processing, and at the same time improve the ability to locate the sound source and the ability to recognize speech (Dornhoffer et al., 2024).

tDCS is a non-invasive neuromodulation technique, using constant, low-intensity direct current therapy to regulate neuronal activity in the cerebral cortex, has been used to treat a variety of mental illnesses and neurological disorders, such as stroke, epilepsy, severe refractory depression, phantom hearing in schizophrenia, and chronic pain, and achieved good results (Kenney-Jung et al., 2019; Chase et al., 2020). From previous studies: the lateralization of stimulation remains a subject of debate, and in the present trial, electrodes were placed on the LTA and rDLPFC. It has been suggested that stimulation of these regions may have different mechanisms of action (Jacquemin et al., 2018). The LTA is involved in speech sound integration and auditory processing, while the rDLPFC plays a crucial role in top-down attentional control and executive functions; the dual-site approach may provide complementary benefits by targeting both sensory and cognitive aspects of hearing rehabilitation. We chose to stimulate both regions sequentially in each treatment. It was found that tDCS improved patients’ auditory function, which is generally consistent with the results of previous studies (Wang et al., 2020). Shekhawat et al. suggested that tDCS could reduce the central gain of tinnitus signals by facilitating the peripheral stimulatory effects of hearing aids, and that tDCS could both facilitate and inhibit cortical activity (Shekhawat et al., 2014). Meanwhile, in another study, transient compensatory auditory stimulation was used to rapidly reduce tinnitus perception, and to further enhance the efficacy, combined HD-tDCS was administered in an attempt to increase the re-adaptation of auditory gain to normal levels and reduce limbic activity (Henin et al., 2016).

On the other hand, MMSE and MoCA scales are authoritative and common scales for clinical assessment of cognitive functions, in which MMSE mainly evaluates patients’ functions related to language, computation, memory, attention, etc., and has higher sensitivity to the group with low education level, and MoCA, which is formulated on the basis of MMSE, has higher sensitivity to the patients with higher education level. The simultaneous use of the two scales reduces the influence of factors such as patients’ education level on the study results. The results of the present study showed that the MoCA and MMSE scores of the patients in both groups were significantly higher after treatment than before treatment, and the MoCA and MMSE scores of the patients in the tDCS group were significantly higher than those in the sham tDCS group after treatment. This suggests that tDCS-assisted auditory rehabilitation training may have a positive effect on the cognitive function of patients with senile hearing impairment. Most previous studies have demonstrated the improving effects of tDCS on cognitive functions, including overall functioning, memory, sustained attention, and executive functioning (Xu et al., 2023).

We further analyzed the correlation between auditory function and cognitive function in elderly patients with hearing impairment, and found that hearing thresholds, HHIE-S, and MoCA and MMSE scores were negatively correlated in elderly patients with hearing impairment. A study by Lancet showed that the risk of dementia would increase by 30% for every 10 dB of hearing loss, and that the risk of dementia would be reduced by 8% if effective interventions for hearing were implemented. Timely hearing intervention for older adults with hearing loss can slow cognitive decline in this population (Livingston et al., 2020; Dawes, 2019).

In addition, the results of our study showed that the total scores of CPA and SF-36 of all patients after treatment were higher than before treatment, and the total score of PRCA-24 was lower than before treatment. The CPA and SF-36 total scores of patients in the tDCS group after treatment were higher than those in the sham tDCS group. It is suggested that tDCS-assisted auditory rehabilitation training may have a positive effect on the communication ability and quality of life of patients with senile hearing impairment. We further counted the adverse reactions in the treatment process of the two groups and found that all patients did not have significant adverse reactions during the treatment process. In our sequence-order subgroup analysis, we found no significant differences in outcomes between patients who received rDLPFC stimulation first versus those who received LTA stimulation first, suggesting that the order effect is minimal in our protocol.

Previous dose–response work has suggested that stimulation intensity is not simply “the stronger the better.” Shekhawat et al. (2013) compared 1 mA versus 2 mA left temporoparietal combined stimulation in 25 patients with chronic tinnitus and found that 2 mA × 20 min achieved more significant transient loudness reduction. Conversely, Shekhawat and Vanneste (2018) reported no significant difference between 1.5 mA and 2 mA for loudness suppression in a 111-case multifactorial optimization trial using right DLPFC stimulation, showing a nonlinear plateau effect. Given that this study used a constant 2 mA, 4 × 1 HD-tDCS, the results should be cautiously extrapolated to other current intensities; future multi-dose designs could further validate the optimal dose window for “auditory-cognitive dual benefit.”

In summary, transcranial direct current stimulation-assisted auditory rehabilitation training may improve cognitive and auditory functions and enhance the quality of life of elderly patients with hearing impairment. However, limitations include a single-center design, non-blinded assessors, power of 0.75, and potential bias. Larger, multicenter studies are needed to validate this result. In addition, the reduction in hearing thresholds may also be related to factors such as improved cooperation after patients become familiar with the audiometric environment and optimization of hearing aid selection while training. This effect applied to both arms and thus unlikely confound between-group comparisons.

## Data Availability

The original contributions presented in the study are included in the article/Supplementary material, further inquiries can be directed to the corresponding author.

## Supplementary material

SUPPLEMENTARY FIGURE S1 Correlation between changes in HHIE-S and MMSE scores.
