# Supplementary material for: Dual improvement of cognitive function and auditory ability in elderly patients with hearing impairment by transcranial direct current stimulation-assisted auditory rehabilitation training
Source: Front Aging Neurosci. 2025 Sep 1;17:1591496. doi: 10.3389/fnagi.2025.1591496 (PMC12434129; doi:10.3389/fnagi.2025.1591496)
Supplement: SUPPLEMENTARY FIGURE S1 — Correlation between changes in HHIE-S and MMSE scores. [file Table_1.docx]

## Complete Supplementary Tables

### Supplementary Table S1 - Bootstrap Mediation Analysis (HHIE-S change → MMSE change)

| **Effect** | **Estimate** | **SE** | **Boot. 95% CI Lower** | **Boot. 95% CI Upper** | **p-value** |
| --- | --- | --- | --- | --- | --- |
| Total effect | 0.15 | 0.05 | 0.05 | 0.26 | 0.003 |
| Direct effect | 0.10 | 0.05 | 0.00 | 0.21 | 0.049 |
| Indirect effect | 0.05 | 0.02 | 0.01 | 0.10 | 0.017 |

**Note:** Mediator = HHIE-S change score; dependent variable = MMSE change score. Estimates derived from 5,000 bias-corrected & accelerated bootstrap resamples.

### Supplementary Table S2 - Sequence-Order Subgroup Analysis (Δ = Post − Pre)

| **Outcome** | **Sequence** | **n** | **Mean Δ** | **SD** | **p-value** |
| --- | --- | --- | --- | --- | --- |
| **Hearing Threshold** | rDLPFC-first | 25 | -9.48 | 3.45 | 0.689 |
|  | LTA-first | 25 | -9.56 | 3.62 |  |
| **HHIE-S** | rDLPFC-first | 25 | -10.08 | 2.87 | 0.776 |
|  | LTA-first | 25 | -9.92 | 3.01 |  |
| **MoCA** | rDLPFC-first | 25 | 2.84 | 1.31 | 0.921 |
|  | LTA-first | 25 | 2.80 | 1.28 |  |
| **MMSE** | rDLPFC-first | 25 | 3.56 | 1.45 | 0.842 |
|  | LTA-first | 25 | 3.48 | 1.52 |  |
| **CPA** | rDLPFC-first | 25 | 12.48 | 4.23 | 0.856 |
|  | LTA-first | 25 | 12.64 | 4.56 |  |
| **PRCA-24** | rDLPFC-first | 25 | -11.12 | 4.98 | 0.723 |
|  | LTA-first | 25 | -10.84 | 5.25 |  |
| **SF-36** | rDLPFC-first | 25 | 8.88 | 4.15 | 0.892 |
|  | LTA-first | 25 | 9.00 | 4.31 |  |

**Note:** p-values from independent-samples t-tests comparing change scores between stimulation-sequence orders. No significant order effects observed for any outcome. Δ = change score (post-treatment minus pre-treatment).

### Supplementary Table S3 - Adverse Events During HD-tDCS Sessions

| **Adverse Effect** | **HD-tDCS (n = 50)** | **Sham (n = 50)** | **χ²** | **p-value** |
| --- | --- | --- | --- | --- |
| Tingling sensation | 6 (12%) | 4 (8%) | 0.44 | 0.505 |
| Mild headache | 3 (6%) | 2 (4%) | 0.21 | 0.647 |
| Burning sensation | 1 (2%) | 0 (0%) | --- | 1.000* |
| Skin redness | 2 (4%) | 1 (2%) | --- | 0.558* |
| Difficulty concentrating | 1 (2%) | 1 (2%) | 0.00 | 1.000 |
| Nausea | 0 (0%) | 0 (0%) | --- | --- |
| Withdrawal due to AE | 0 (0%) | 0 (0%) | --- | --- |
| **Total subjects with any AE** | **10 (20%)** | **7 (14%)** | **0.63** | **0.426** |

**Note:** Values are counts with percentages in parentheses. *Fisher's exact test used for sparse cells. AE = adverse events. All adverse events were mild and transient, resolving within 30 minutes after stimulation cessation.

### Supplementary Table S4 - Two-Way Repeated-Measures ANOVA (Group × Time)

| **Outcome** | **Factor** | **df** | **F** | **p-value** | **Partial η²** |
| --- | --- | --- | --- | --- | --- |
| **Hearing Threshold** | Time | 1, 98 | 134.7 | <0.001 | 0.58 |
|  | Group | 1, 98 | 8.9 | 0.004 | 0.08 |
|  | Interaction | 1, 98 | 5.4 | 0.022 | 0.05 |
| **HHIE-S** | Time | 1, 98 | 186.4 | <0.001 | 0.66 |
|  | Group | 1, 98 | 11.3 | 0.001 | 0.10 |
|  | Interaction | 1, 98 | 7.8 | 0.006 | 0.07 |
| **MoCA** | Time | 1, 98 | 42.3 | <0.001 | 0.30 |
|  | Group | 1, 98 | 4.1 | 0.046 | 0.04 |
|  | Interaction | 1, 98 | 4.8 | 0.031 | 0.05 |
| **MMSE** | Time | 1, 98 | 58.2 | <0.001 | 0.37 |
|  | Group | 1, 98 | 9.8 | 0.002 | 0.09 |
|  | Interaction | 1, 98 | 6.5 | 0.012 | 0.06 |
| **CPA** | Time | 1, 98 | 98.3 | <0.001 | 0.50 |
|  | Group | 1, 98 | 6.8 | 0.011 | 0.07 |
|  | Interaction | 1, 98 | 8.2 | 0.005 | 0.08 |
| **PRCA-24** | Time | 1, 98 | 112.5 | <0.001 | 0.53 |
|  | Group | 1, 98 | 1.5 | 0.224 | 0.02 |
|  | Interaction | 1, 98 | 1.3 | 0.257 | 0.01 |
| **SF-36** | Time | 1, 98 | 84.7 | <0.001 | 0.46 |
|  | Group | 1, 98 | 5.9 | 0.017 | 0.06 |
|  | Interaction | 1, 98 | 7.1 | 0.009 | 0.07 |

**Note:** df = degrees of freedom; Partial η² ≥ 0.14 indicates a large effect size, 0.06-0.14 indicates medium effect size, 0.01-0.06 indicates small effect size.
